# The 15-year national trends of endocrine cancers incidence among Iranian men and women; 2005–2020

**DOI:** 10.1038/s41598-023-34155-2

**Published:** 2023-05-10

**Authors:** Narges Zargar Balajam, Amir-Hossein Mousavian, Ali Sheidaei, Kimiya Gohari, Seyed Mohammad Tavangar, Ali Ghanbari-Motlagh, Afshin Ostovar, Gita Shafiee, Ramin Heshmat

**Affiliations:** 1 Chronic Diseases Research Center, Endocrinology and Metabolism Population Sciences Institute, Tehran University of Medical Sciences, Tehran, Iran; 2 Department of Epidemiology and Biostatics, School of Public Health, Tehran University of Medical Sciences, Tehran, Iran; 3 Department of Biostatistics, Faculty of Medicine Sciences, Tarbiat Modares University, Tehran, Iran; 4 Department of Radiotherapy, School of Medicine, Shahid Beheshti University of Medical Sciences, Tehran, Iran; 5 Osteoporosis Research Center, Endocrinology and Metabolism Clinical Sciences Institute, Tehran University of Medical Sciences, Tehran, Iran

**Keywords:** Cancer, Endocrinology, Medical research

## Abstract

Cancer is one of the important health problems in Iran, which is considered as the third cause of death. Endocrine cancers are rare but mostly curable. Thyroid cancer, the most common endocrine tumors, includes about one percent of malignant cancer. In this study, we examined the 15-year national trend of endocrine cancer incidence in Iranian men and women. The data in each province were evaluated based on age, gender, and cancer type according to International Classification of Disease Codes version 10 (ICD-10) from 2005 to 2020 in Iran. All data were obtained from the reports of the Statistics Center of Iran (SCI), 6 phases of the step-by-step approach to monitoring the risk factors of chronic diseases over 18 years old (STEPs), and 3 periods of the CASPIAN study (survey of non-communicable diseases in childhood and adolescence). Statistical analyzes and graph generation were done using R statistical software. Poisson regression with mixed effects was used for data modeling and incidence rate estimation. The incidence of thyroid gland malignancy is higher in women than in men. On the other hand, the incidence of adrenal gland cancer is slightly higher in men than in women. The same pattern is observed for other endocrine neoplasms and related structures. The incidence rate of these types of cancers has generally increased from 2005 to 2020 in Iran. This increase is more in women than in men. In addition, in the middle of the country, there is a strong region in terms of the occurrence of these types of cancers. The incidence rate in these provinces is relatively higher for both sexes and all studied periods. We conducted a study to observe the changing trends for various types of endocrine cancers over 15 years in men and women. Considering the increasing trend of thyroid cancers in Iran, therefore, creating essential policies for the management of these types of cancers for prevention, rapid diagnosis, and, timely treatment is particularly important.

## Introduction

Despite significant progress in cancer prevention and diagnosis, it is still considered one of the major health problems in societies and is responsible for nearly one in six global deaths nowadays^1^. Also, cancer is known as the second cause of death in the United States with the prediction of more than 1.9 million new cases and 609,360 deaths per day in 2022^2^. According to the assessments of the cancer incidence in Iran in 2020 (152.7 per 100,000), the intermediate risk for cancer can be considered in Iran to compare of other countries in the world^3,4^. Based on the GLOBOCAN estimate and excluding the continuous changes in cancer-specific incidence rates over time, the overall growth rate of new cancer cases in Iran is estimated to be 17.3% until 2025^5^. In a study by Golestan Province Population-Based Cancer Registration Center (GPCR) in Iran, the rate of increase in the number of new cancer cases except non-melanoma skin cancers [NMSCs] is predicted to be 61.3% between 2016 and 2025^6^.

A group of diseases characterized by uncontrolled cell proliferation of hormone-producing glands in the endocrine system are identified as endocrine cancers. Although endocrine cancers are fairly uncommon, they are an important group of treatable cancers^7^. Generally, less than 1% of all malignancies are related to thyroid cancer. In addition, Thyroid cancer is the most common type of endocrine cancer with an estimated approximately 11,860 and 31,940 new cases in men and women in the United States in 2022 respectively^2,8^. More than 90% of thyroid cancers are differentiated thyroid cancer (DTC), which includes papillary thyroid cancer (PTC) and follicular thyroid cancer (FTC)^8^. Also, medullary thyroid carcinoma (MTC) comprises 5–10%, and anaplastic thyroid carcinoma rarely (1–2%) of all thyroid cancers^7^. Recent research by US Preventive Services Task Force has revealed that thyroid cancer is currently decreasing in men and women at a rate of 2.5% per year from 2014 to 2018^9^. On the other hand, the annual incidence rate of DTC has been growing in current years, which is three times higher in women than in men^10–12^. The 5-year survival rate for this type of thyroid cancer (DTC) has been reported to be approximately 93% and 88% in women and men, respectively^11^.

In a study in four cities of Iran, the population of endocrinological cancers was investigated between 1996 and 2000. 82.7% of patients had papillary type of thyroid cancer. The incidence rate of this cancer in males (0.627) was lower than that in females (1.59)^13^.

Thyroid cancer was the third most common malignancy among Iranian women between 2014 and 2016, with a prevalence rate of 8.2%, which is expected to increase to 13.8% by 2025^14^. According to WHO International Cancer Research Agency estimates, thyroid cancer with 3172 (5.2%) new cases in women has been recorded as the fifth most common cancer in the world in 2020^15^.

In recent years, several studies have been conducted on endocrine cancer in Iran. It is particularly important to investigate the prevalence and occurrence of various types of cancer as one of the most important diseases that people are dealing with and that imposes a large financial and social burden on society and families. Therefore, this study was conducted by examining the trend of endocrine cancer between 2005 and 2020 in Iran in order to prevent and identify strategies for managing this type of cancer in the country.

## Methods

### Data sources

#### Cancer incidence

The ministry of health and medical education has been performing a population-based registry for incident cases of cancer in Iran. The data is validated and available for 2008 to 2010, 2014, and 2015 now. The information at the individual level consists of age, sex, province of residence, and international classification of disease version 10 (ICD-10) codes.

We targeted to explore the principal ICD10 codes C73 (Malignant neoplasm of the thyroid gland), C74 (Malignant neoplasm of the adrenal gland), and C75 (Malignant neoplasm of other endocrine glands and related structures) and their subcategory in this study. The malignant neoplasm of the adrenal gland consists of C74.0 (Cortex of adrenal gland), C74.1 (Medulla of the adrenal gland), and C74.9 (Adrenal gland, unspecified). On the other hand, malignant neoplasm of other endocrine glands and related structures includes C75.0 (Parathyroid gland), C75.1 (Pituitary gland), C75.2 (Craniopharyngeal duct), C75.3 (Pineal gland), C75.4 (Carotid body), C75.5 (Aortic body and other paraganglia), C75.8 (Pluriglandular involvement, unspecified), and C75.9 (Endocrine gland, unspecified). According to the low frequency in subcategories which leads to decreased precision of estimations and non-convergence of the models, we preferred to work on the root ICD-10 codes^16^. In this study, age groups from 15 to over 75 were categorized with a 10-year age difference between them^17,18^.

#### Population and covariates data

The population data for each age-sex group at the provincial level were extracted from the Statistical Center of Iran (SCI) reports. In general, the main source of information for this study was obtained from the cancer registration system of the entire country of Iran. The actual data was obtained from the population and housing census in 2001, 2006, 2011, and 2016. In addition, the SCI extrapolated the population in the subgroups by the year 2350 using the cohort-component method. The information is publicly available online on the SCI website^19,20^.

Based on the relevance and availability of data, a list of potential covariates was selected for the modeling section. Our target population is individuals more than 15 years old covered by two nationally representative surveys. According to the World Health Organization (WHO) guideline, these surveys are conducted regularly in Iran's health systems^21^.

The stepwise approach to chronic disease risk factor surveillance known as STEPs focuses on risk factors of non-communicable diseases in adults more than 18 years old. We used all 6 phases of this survey conducted in 2005, 2007, 2008, 2009, 2011, and 2016^22^.

In order to cover the population under this age, we add information of childhood and adolescent surveillance and prevention of adult non-communicable diseases (CASPIAN study). This survey follows the WHO, global school-based student health survey (GHSH) instructions and covers the adolescent population at school age^23^. Data for CASPIAN-III (2009–2010), CASPIAN-IV (2011–2012), and CASPIAN-V (2015) were used^24^.

Finally, the ratio of urbanization was used as an indicator to differentiate urban–rural lifestyles. This variable is derived from the population dataset reported by SCI^19^.

A series of potentially related covariates were extracted to cooperate in modeling. As the registration system only records incident information, we designed an ecological study and worked on aggregated data by age, sex, province, and year. In this manner, the individual level calculated covariates were aggregated to construct a representative value for desired combinations. Unavailable actual data were assessed by nonparametric spline smoothing method using R statistical software. In this way, a uniform cubic spline was calculated using the Hyman filter. Finally, covariates variables were calculated and smoothed at all levels of province-age-sex throughout the year^25^.

#### Statistical modeling

We defined the total number of new cases due to neoplasm types of interest as the outcome variable in this study. In this regard, Poisson regression with mixed effects was used to model the data and estimate the incidence rate^26^. Appropriate separate models were designed, for each type of malignant neoplasm. The logarithm of the number of new cases against the fixed effect of covariates as well as the fixed effects of age groups as dummy variables was modeled. Correlation between incident cases across times with random effect of year and unknown causes of variations within provinces were obtained by the random effect of the province. Finally, the offset variable was considered in the at-risk population model. For better data presentation, a direct approach was used for age standardization according to reference population national in 2020. In addition, the Average annual percent of changes (AAPC) was calculated to exhibit a better understanding of temporal changes. All statistical analysis and graph generations were conducted in R statistical software environment.

#### Model building and validation

After randomly dividing the data set into five subsets, at each stage, four parts of these subsets were applied for building modeling and the other for checking the results. The root (the squared error) was used to assess the models. An analogous approach was used to select the best definition of age groups.

### Ethical consideration

This study has the permission of the Ethics Committee of Tehran University of Medical Sciences and informed consent of the participants. Approval ID: IR.TUMS.EMRI.REC.1397.051.

## Results

**Figure 1:**
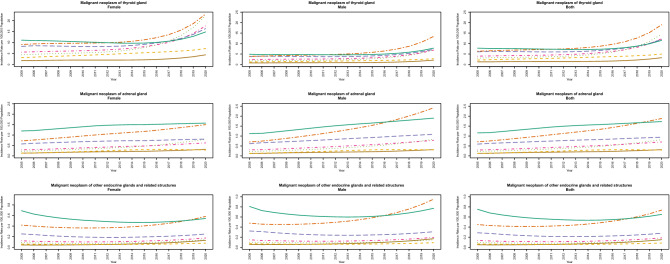
Age specific trends of incidence rate of malignant neoplasms of thyroid and other endocrine glands in 100,000 population.

The gender and time distribution of age-specific and all-ages estimations of incidence rates of endocrine neoplasms are presented in Fig. 1. The incidence of malignant neoplasm of the thyroid gland is relatively higher in females [13.21 (12.77–13.65) in 2020] than in males [5.17 (4.72–5.62) in 2020]. On the other hand, the incidence of malignant neoplasm of the adrenal gland is slightly higher in males [0.7 (0.13–1.68)] than in females [0.61 (0.08–1.59) in 2020]. The same pattern is observed for the malignant neoplasm of other endocrine glands and related structures. There are increasing trends for neoplasms of the thyroid gland and adrenal gland in the study period.

The incidence rates 2.8 (2.64–2.96) and 0.24 (0–1.56) in 2005 increased to 9.23 (8.78–9.67) and 0.66 (0.1–1.63) in 2020 for these neoplasms, respectively. Although, as a general pattern, the incidence of the malignant neoplasm of the thyroid gland increased across age groups, we see elevated rates in more inferior age groups in recent years. For instance, the incidence rates of this type of neoplasm in 2020 are estimated to be 14.41 (14.08–14.75) and 18.97 (17.48–20.47) in 100,000 population in age groups 35–44 and 65–74 years old, respectively.

**Figure 2:**
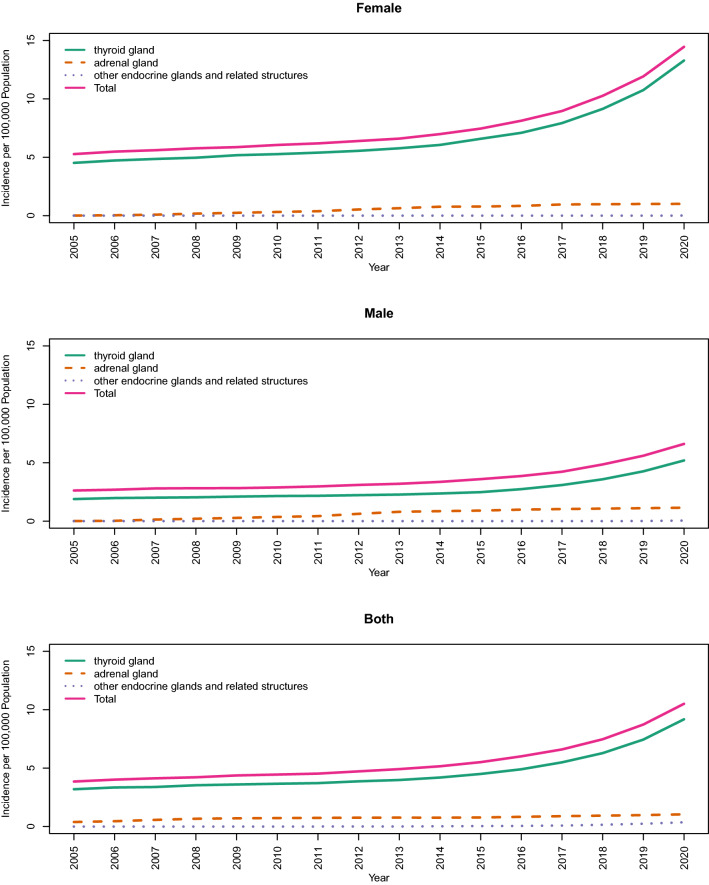
Age standardized incidence rate of malignant neoplasms of thyroid and other endocrine glands in 100,000 population.

**Figure 3:**
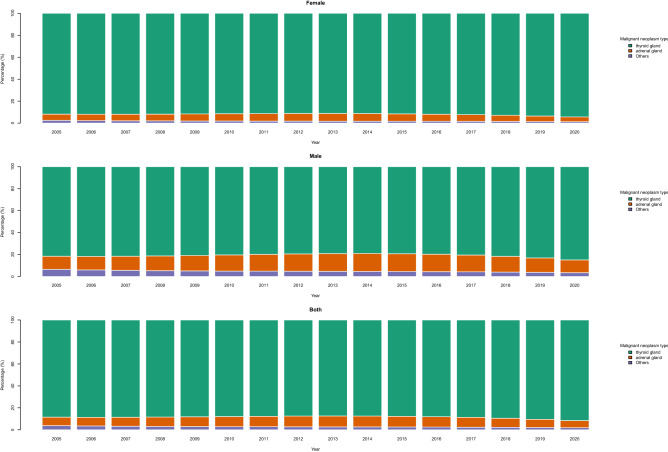
Percentage of each type of malignant neoplasms from the total malignant neoplasms of thyroid and other endocrine glands.

The trend of age-standardized incidence rates of endocrine neoplasms is depicted in Fig. 2. According to this figure, the incidence rates increased from 2005 to 2020 overall. However, the increasing slope elevated after 2015. This boost seems to be higher in females than males. The proportions of all categories from the total new cases of endocrine neoplasms are practically constant over time. The only exception is the share of the adrenal gland, which increases barely between 2010 to 2015 and then declines again (Fig. 3).

**Figure 4:**
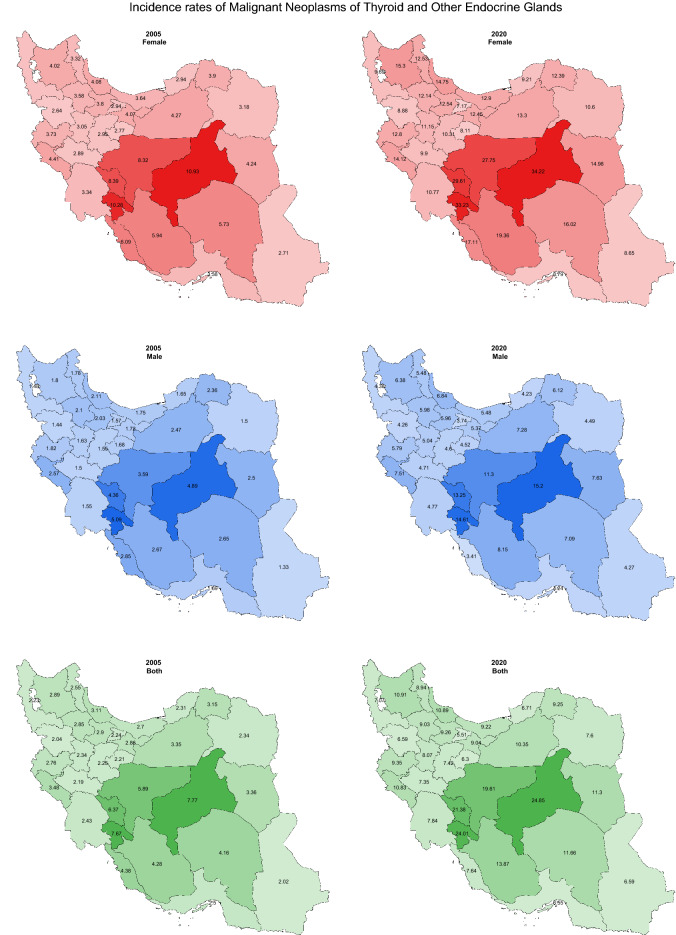
Geographical distribution of malignant neoplasms of thyroid and other endocrine glands incidence rates per 100,000 population in 2005 and 2020.

The subnational analysis results in heterogeneity between Iran provinces according to new cases of endocrine neoplasms. In addition, there is a hotspot in the country's middle, including Isfahan, Yazd, Chahar Mahaal and Bakhtiari, and Kohgiluyeh and Boyer-Ahmad. The incidence rates are relatively higher in these provinces for both sexes and all the periods of study (Fig. 4).

The lowest AAPC of incidence rate per 100,000 population for malignant neoplasm of the thyroid gland is related to Bushehr which increased from 3.57 in 2005 to 6.60 in 2020. It means the incidence rate increased 4.27% annually on average. On the other hand, the incidence rate of this type of neoplasm increased 9.58% annually on average in Azerbaijan, East from 2005 to 2020 as the most increase in the country (Table 1).

**Table 1:** Sub-national variation of malignant neoplasm of thyroid gland across years and provinces (all ages incidence rate per 100,000 female population).

Province	Female					Male					Both				
	Year				AAPC	Year				AAPC	Year				AAPC
	2005	2010	2015	2020		2005	2010	2015	2020		2005	2010	2015	2020	
Alborz	2.35	2.57	3.15	6.14	6.78	0.96	1.04	1.25	2.50	6.76	1.64	1.79	2.21	4.37	6.95
Ardabil	2.85	3.62	5.08	11.38	9.83	1.25	1.49	1.94	4.20	8.64	2.05	2.55	3.49	7.73	9.42
Azerbaijan, East	3.85	4.86	6.73	14.89	9.60	1.62	1.98	2.66	5.90	9.18	2.72	3.41	4.70	10.46	9.58
Azerbaijan, West	2.68	3.22	4.24	8.99	8.57	1.14	1.32	1.68	3.55	8.04	1.90	2.26	2.97	6.30	8.49
Bushehr	5.28	6.21	7.81	15.55	7.63	2.05	2.14	1.92	2.60	1.70	3.57	3.99	4.20	6.60	4.27
Chahar Mahaal and Bakhtiari	7.29	9.02	12.34	26.88	9.26	3.16	3.72	4.80	10.17	8.28	5.22	6.36	8.55	18.47	8.96
Fars	5.71	6.92	9.08	18.86	8.45	2.42	2.85	3.64	7.56	8.07	4.04	4.87	6.38	13.32	8.44
Gilan	3.64	4.56	6.24	13.65	9.38	1.62	1.94	2.55	5.52	8.70	2.64	3.27	4.43	9.68	9.23
Golestan	2.48	2.96	3.90	8.24	8.53	1.11	1.25	1.54	3.13	7.33	1.80	2.11	2.72	5.68	8.12
Hamadan	2.60	3.28	4.53	10.00	9.57	1.13	1.36	1.76	3.76	8.50	1.87	2.32	3.14	6.85	9.22
Hormozgan	2.84	3.19	3.95	7.81	7.15	1.14	1.27	1.55	3.09	7.01	1.96	2.22	2.75	5.48	7.27
Ilam	3.28	4.04	5.42	11.49	8.88	1.38	1.65	2.12	4.46	8.31	2.31	2.83	3.77	8.00	8.79
Isfahan	8.08	9.76	12.92	27.21	8.60	3.34	3.93	5.06	10.68	8.24	5.65	6.80	8.98	19.04	8.60
Kerman	5.33	6.13	7.64	15.23	7.42	2.23	2.50	3.06	6.14	7.17	3.75	4.30	5.37	10.80	7.48
Kermanshah	3.34	4.11	5.53	11.90	9.00	1.41	1.68	2.20	4.72	8.59	2.36	2.89	3.87	8.37	8.98
Khorasan, North	3.07	3.75	4.97	10.54	8.75	1.40	1.61	1.98	4.02	7.47	2.25	2.69	3.48	7.27	8.30
Khorasan, Razavi	3.03	3.65	4.85	10.27	8.65	1.33	1.54	1.96	4.09	7.97	2.18	2.60	3.42	7.23	8.49
Khorasan, South	3.24	4.06	5.63	12.50	9.60	1.39	1.68	2.22	4.79	8.77	2.31	2.87	3.92	8.65	9.38
Khuzestan	3.10	3.71	4.87	10.23	8.46	1.30	1.52	1.95	4.12	8.20	2.18	2.61	3.42	7.24	8.51
Kohgiluyeh and Boyer-Ahmad	9.30	11.25	14.85	31.16	8.56	4.00	4.73	5.99	12.19	7.87	6.64	8.01	10.48	21.77	8.40
Kurdistan	2.18	2.68	3.61	7.84	9.07	0.93	1.11	1.43	3.05	8.38	1.55	1.89	2.53	5.46	8.91
Lorestan	2.46	3.05	4.13	8.91	9.11	1.05	1.26	1.65	3.53	8.63	1.75	2.15	2.90	6.27	9.06
Markazi	2.53	3.16	4.31	9.33	9.26	1.09	1.29	1.66	3.50	8.27	1.81	2.22	2.97	6.39	8.95
Mazandaran	3.41	4.24	5.75	12.35	9.12	1.49	1.76	2.27	4.84	8.34	2.45	3.00	4.01	8.63	8.92
Qazvin	3.14	3.83	5.17	11.10	8.94	1.32	1.55	2.01	4.29	8.38	2.21	2.68	3.58	7.71	8.85
Qom	1.88	2.23	2.93	6.17	8.40	0.78	0.88	1.12	2.33	7.77	1.32	1.54	2.01	4.23	8.27
Semnan	3.16	3.84	5.10	10.83	8.73	1.30	1.55	2.02	4.33	8.53	2.21	2.68	3.57	7.65	8.82
Sistan and Baluchistan	2.44	2.91	3.83	8.05	8.46	1.04	1.22	1.61	3.50	8.63	1.73	2.07	2.75	5.91	8.71
Tehran	3.97	4.62	5.94	12.24	7.97	1.62	1.89	2.42	5.11	8.14	2.76	3.24	4.21	8.80	8.21
Yazd	9.95	12.08	15.77	32.07	8.26	3.93	4.65	6.03	12.67	8.29	6.80	8.23	10.83	22.52	8.47

The incidence rates for the other two types of endocrine categories are presented in Tables 2 and 3. According to these results, the highest and lowest increases were related to Azerbaijan, East, and Bushehr, respectively. As an exception to the overall pattern of the country, the AAPC of Bushehr for malignant neoplasm of other endocrine glands and related structures is the negative value for males and both sexes with values − 2.63% and − 0.73%.

**Table 2:** Sub-national variation of malignant neoplasm of adrenal gland across years and provinces (all ages incidence rate per 100,000 female population).

Province	Female					Male					Both				
	Year				AAPC	Year				AAPC	Year				AAPC
	2005	2010	2015	2020		2005	2010	2015	2020		2005	2010	2015	2020	
Alborz	0.41	0.54	0.66	0.80	4.52	0.40	0.54	0.73	0.97	6.06	0.41	0.54	0.69	0.88	5.30
Ardabil	0.26	0.39	0.54	0.75	7.38	0.27	0.40	0.58	0.83	7.82	0.26	0.39	0.56	0.79	7.62
Azerbaijan, East	0.10	0.15	0.21	0.30	7.23	0.10	0.16	0.24	0.35	8.42	0.10	0.16	0.23	0.32	7.84
Azerbaijan, West	0.22	0.32	0.42	0.55	6.22	0.23	0.33	0.46	0.65	7.30	0.23	0.32	0.44	0.60	6.77
Bushehr	0.56	0.77	0.97	1.20	5.26	0.51	0.67	0.67	0.61	1.25	0.53	0.72	0.79	0.79	2.72
Chahar Mahaal and Bakhtiari	0.85	1.24	1.70	2.29	6.82	0.88	1.28	1.84	2.58	7.47	0.86	1.26	1.77	2.44	7.16
Fars	0.15	0.21	0.28	0.36	6.20	0.15	0.21	0.31	0.42	7.40	0.15	0.21	0.29	0.39	6.81
Gilan	0.32	0.48	0.65	0.89	6.99	0.34	0.51	0.74	1.06	7.92	0.33	0.49	0.69	0.97	7.47
Golestan	0.26	0.37	0.49	0.64	6.14	0.28	0.39	0.53	0.72	6.57	0.27	0.38	0.51	0.68	6.37
Hamadan	0.33	0.48	0.67	0.92	7.14	0.34	0.50	0.73	1.03	7.71	0.33	0.49	0.70	0.97	7.44
Hormozgan	0.38	0.50	0.63	0.77	4.81	0.36	0.50	0.68	0.90	6.26	0.37	0.50	0.65	0.83	5.55
Ilam	0.84	1.21	1.62	2.14	6.46	0.83	1.24	1.77	2.47	7.51	0.84	1.23	1.70	2.30	7.00
Isfahan	0.17	0.24	0.32	0.42	6.34	0.16	0.24	0.35	0.49	7.57	0.17	0.24	0.33	0.45	6.96
Kerman	0.31	0.43	0.53	0.66	5.14	0.31	0.44	0.59	0.80	6.47	0.31	0.43	0.56	0.73	5.82
Kermanshah	0.27	0.39	0.53	0.71	6.60	0.27	0.40	0.59	0.84	7.80	0.27	0.40	0.56	0.77	7.21
Khorasan, North	0.56	0.81	1.07	1.40	6.33	0.60	0.86	1.18	1.59	6.67	0.58	0.83	1.12	1.50	6.52
Khorasan, Razavi	0.10	0.14	0.19	0.25	6.40	0.10	0.15	0.21	0.30	7.33	0.10	0.15	0.20	0.27	6.87
Khorasan, South	0.65	0.97	1.33	1.84	7.14	0.67	1.00	1.46	2.09	7.95	0.66	0.98	1.40	1.97	7.56
Khuzestan	0.18	0.26	0.34	0.44	6.17	0.18	0.26	0.37	0.53	7.49	0.18	0.26	0.36	0.48	6.84
Kohgiluyeh and Boyer-Ahmad	0.61	0.87	1.15	1.49	6.14	0.62	0.91	1.29	1.73	7.08	0.62	0.89	1.21	1.61	6.62
Kurdistan	0.28	0.40	0.55	0.73	6.65	0.28	0.42	0.60	0.85	7.59	0.28	0.41	0.57	0.79	7.13
Lorestan	0.28	0.41	0.56	0.75	6.71	0.28	0.43	0.62	0.88	7.85	0.28	0.42	0.59	0.82	7.29
Markazi	0.26	0.38	0.52	0.70	6.84	0.27	0.39	0.56	0.79	7.48	0.26	0.39	0.54	0.74	7.18
Mazandaran	0.16	0.23	0.32	0.43	6.77	0.17	0.24	0.35	0.50	7.60	0.16	0.24	0.33	0.46	7.20
Qazvin	0.38	0.54	0.73	0.97	6.53	0.37	0.55	0.79	1.12	7.58	0.38	0.54	0.76	1.04	7.07
Qom	0.69	0.96	1.26	1.64	5.99	0.67	0.95	1.34	1.85	6.97	0.68	0.95	1.30	1.75	6.50
Semnan	0.76	1.08	1.44	1.89	6.30	0.74	1.10	1.58	2.25	7.71	0.75	1.09	1.51	2.07	7.02
Sistan and Baluchistan	0.19	0.27	0.36	0.47	6.08	0.20	0.29	0.42	0.61	7.83	0.19	0.28	0.39	0.53	6.95
Tehran	0.07	0.09	0.12	0.16	6.03	0.07	0.10	0.14	0.20	7.69	0.07	0.09	0.13	0.18	6.87
Yazd	0.78	1.11	1.45	1.83	5.89	0.73	1.07	1.54	2.15	7.51	0.75	1.09	1.50	1.99	6.71

**Table 3:** Sub-national variation of malignant neoplasm of other endocrine glands and related structures across years and provinces (All ages incidence rate per 100,000 female population).

Province	Female					Male					Both				
	Year				AAPC	Year				AAPC	Year				AAPC
	2005	2010	2015	2020		2005	2010	2015	2020		2005	2010	2015	2020	
Alborz	0.18	0.15	0.16	0.23	1.81	0.21	0.17	0.20	0.28	2.06	0.19	0.16	0.18	0.25	1.91
Ardabil	0.21	0.20	0.25	0.40	4.39	0.27	0.24	0.29	0.45	3.60	0.24	0.22	0.27	0.42	3.97
Azerbaijan, East	0.06	0.06	0.07	0.12	4.34	0.08	0.07	0.09	0.14	4.22	0.07	0.07	0.08	0.13	4.26
Azerbaijan, West	0.09	0.08	0.10	0.15	3.39	0.11	0.10	0.12	0.18	3.19	0.10	0.09	0.11	0.17	3.26
Bushehr	0.26	0.23	0.26	0.36	2.35	0.29	0.23	0.20	0.20	− 2.63	0.28	0.23	0.22	0.25	− 0.73
Chahar Mahaal and Bakhtiari	0.25	0.23	0.28	0.44	3.85	0.32	0.28	0.34	0.50	3.25	0.28	0.26	0.31	0.47	3.52
Fars	0.09	0.08	0.09	0.14	3.47	0.11	0.10	0.11	0.17	3.36	0.10	0.09	0.10	0.16	3.38
Gilan	0.12	0.11	0.14	0.21	4.09	0.15	0.14	0.17	0.26	3.74	0.14	0.13	0.15	0.24	3.89
Golestan	0.20	0.18	0.21	0.32	3.25	0.26	0.23	0.25	0.37	2.46	0.23	0.20	0.23	0.35	2.83
Hamadan	0.12	0.12	0.14	0.23	4.19	0.16	0.14	0.17	0.26	3.51	0.14	0.13	0.16	0.24	3.82
Hormozgan	0.16	0.13	0.15	0.21	1.97	0.19	0.16	0.18	0.25	2.15	0.17	0.15	0.16	0.23	2.04
Ilam	0.30	0.27	0.33	0.49	3.47	0.36	0.33	0.39	0.58	3.27	0.33	0.30	0.36	0.53	3.35
Isfahan	0.07	0.06	0.08	0.12	3.61	0.08	0.08	0.09	0.14	3.53	0.08	0.07	0.08	0.13	3.54
Kerman	0.09	0.08	0.09	0.13	2.39	0.11	0.09	0.11	0.15	2.43	0.10	0.09	0.10	0.14	2.38
Kermanshah	0.11	0.10	0.12	0.19	3.69	0.14	0.12	0.15	0.23	3.60	0.12	0.11	0.14	0.21	3.62
Khorasan, North	0.27	0.24	0.29	0.44	3.37	0.36	0.31	0.35	0.51	2.49	0.31	0.28	0.32	0.47	2.91
Khorasan, Razavi	0.05	0.05	0.06	0.08	3.71	0.06	0.06	0.07	0.10	3.35	0.06	0.05	0.06	0.09	3.50
Khorasan, South	0.35	0.33	0.41	0.64	4.14	0.44	0.40	0.48	0.74	3.69	0.40	0.36	0.45	0.69	3.89
Khuzestan	0.06	0.06	0.07	0.10	3.42	0.08	0.07	0.08	0.12	3.42	0.07	0.06	0.07	0.11	3.39
Kohgiluyeh and Boyer-Ahmad	0.37	0.33	0.39	0.58	3.18	0.46	0.41	0.48	0.69	2.86	0.41	0.37	0.43	0.64	2.99
Kurdistan	0.18	0.17	0.20	0.31	3.71	0.23	0.20	0.24	0.37	3.39	0.20	0.18	0.22	0.34	3.52
Lorestan	0.14	0.13	0.16	0.24	3.78	0.17	0.16	0.19	0.29	3.64	0.16	0.14	0.17	0.26	3.68
Markazi	0.16	0.14	0.18	0.27	3.89	0.19	0.18	0.21	0.31	3.29	0.17	0.16	0.19	0.29	3.56
Mazandaran	0.07	0.06	0.08	0.12	3.90	0.09	0.08	0.10	0.14	3.48	0.08	0.07	0.09	0.13	3.66
Qazvin	0.28	0.25	0.31	0.47	3.58	0.34	0.31	0.37	0.55	3.37	0.31	0.28	0.34	0.51	3.45
Qom	0.19	0.17	0.20	0.30	3.07	0.23	0.20	0.23	0.35	2.80	0.21	0.19	0.22	0.32	2.91
Semnan	0.36	0.32	0.38	0.58	3.33	0.43	0.39	0.46	0.70	3.47	0.39	0.35	0.42	0.64	3.38
Sistan and Baluchistan	0.08	0.07	0.09	0.13	3.21	0.10	0.09	0.11	0.17	3.64	0.09	0.08	0.10	0.15	3.38
Tehran	0.03	0.03	0.03	0.05	3.87	0.04	0.03	0.04	0.06	3.97	0.03	0.03	0.04	0.06	3.88
Yazd	0.21	0.18	0.22	0.31	2.94	0.24	0.21	0.25	0.38	3.29	0.22	0.20	0.24	0.35	3.10

## Discussion

This study aimed to investigate the national trend of endocrine cancer in men and women between 2005 and 2020. The total number of endocrine cancers detected, regardless of sex, has increased during these years. Also, the incidence of endocrine cancers has been increasing in these times, which thyroid cancers have displayed the highest occurrence among women and men. On the other hand, the growing incidence trend of this cancer has been slow until 2015, and then upsurges considerably. Despite the same pattern for both sexes in this type of cancer, it is more severe in women.

This pattern is similar to the studies conducted outside of Iran^27,28^. Indeed, by expanding the use of diagnostic procedures and imaging, most notably fine-needle aspiration and ultrasound, the number of detected cancers, especially small and asymptomatic nodules have amplified^29,30^. Part of this remarkable increasing incidence is considered due to overdiagnosis and excessive use of measurement methods^28,31,32^. Although the role of diagnosis in the process of thyroid cancer is momentous, it is not solely responsible for the increased incidence rate since a huge proportion of detected nodules are large nodules^28,32^. Furthermore, if this enhancement was only due to technological advancements and detection trials, would not be increased faster in developing countries compared with the developed^33^.

Environmental factors could result in the incidence of thyroid cancer. Increased head and neck imaging followed by exposure to ionizing radiation affect the incidence of thyroid cancer^34^. Obesity and smoking are other risk factors associated with a higher incidence of thyroid cancer. Studies display that normal and underweight are less likely to develop thyroid cancer while overweight and obese people have more chance of developing thyroid cancer^35,36^.

Although there is a clear increment in the incidence of adrenal gland cancer, it does not follow the pattern of thyroid cancer and has a mild upgoing trend. The prevalence of adrenal cancer has slightly increased over the duration of 2005–2020. There is no noteworthy difference between the incidence rate of males and females. Despite the increased number of people detected with this cancer, however, the proportion of adrenal cancer compared with other endocrine cancers has reduced. Indeed, the prevalence of adrenal cancer in Iran is similar to other countries (average 2 cases per 100,000 annually)^37,38^.

In terms of Adrenocortical Cancer, women get it more than men. According to studies, heavy smoking in men and consumption of oral contraceptives in women are considered as risk factors that increase this cancer^38–40^.

In a comprehensive study in 2016 by Taghavi and et al., the national and sub national trend of the prevalence of thyroid cancer in the Iranian population from 1990 to 2010 was investigated. They used linear and logistic regression model for modeling and prediction. Therefore, they stated that the prevalence of thyroid cancer has increased especially during the years 2002–2010, and this ratio in women was 2.5 times more than of men. In addition, the four provinces of Iran including Isfahan, Yazd, Tehran and Qazvin respectively showed the highest prevalence during this period^41^.

Finally, no other study has demonstrated the prevalence trend of these cancers in Iran. The strength of this study is the duration of data collection, which reveals the incidence trend for each gender and for both age-standardized and age-specific groups. We had to use statistical modeling for data synthesis resulted in incomplete data in our registry. Another limitation we faced, was that the type of thyroid cancers detected are unknown. Also, the prevalence of adrenal cancers is not so high that it requires immediate action, though it should not be forgotten.

## Conclusion

Nowadays among all types of endocrine cancers, the incidence of thyroid and adrenal cancers is growing in both men and women. Also, the prevalence rate of these malignancies is higher among the central provinces the exact cause of which needs to be investigated and studied. Although part of this incidence is a result of overdiagnosis, the number of detected neoplasms is too high to ignore. The uncertainty toward these problems reminds us that there are much more to learn and discover. In addition, more studies should be done in order to increase awareness in identifying events, and environmental, internal factors that may growth the risk of such cancers.

## Data Availability

We would like to inform all the reviewers that our data, analytic methods, and study materials are available upon request, by contacting our corresponding author.
